# Ebola Virus Disease has Features of Hemophagocytic Lymphohistiocytosis Syndrome

**DOI:** 10.3389/fmed.2015.00004

**Published:** 2015-02-04

**Authors:** Andre J. A. M. van der Ven, Mihai G. Netea, Jos W. M. van der Meer, Quirijn de Mast

**Affiliations:** ^1^Department of Internal Medicine, Radboud University Medical Centre, Nijmegen, Netherlands

**Keywords:** Ebola virus, macrophage activation, anakinra, cytokines, ferritins

The 2014 epidemic of Ebola virus disease (EVD) in several West African countries has mortality rates of up to 70%, as reported by WHO. The exponential increase in the number of infections and deaths, coupled with the absence of specific preventive and therapeutic strategies, represents one of the biggest global health challenges of this millenium. Insights in the pathophysiology and treatment of EVD are thus urgently needed. In this paper, we postulate that EVD has the characteristics of hemophagocytic lymphohistiocytosis (HLH) syndrome (syn., macrophage activation syndrome). The identification of HLH as part of severe EVD, however, brings the possibility for a pathophysiologically targeted approach of therapy; special promise is represented by cytokine-directed therapy in the form of recombinant interleukin-1 receptor antagonist (anakinra). Treatment with anakinra has the advantage to interrupt the deleterious IL-1-mediated hyperinflammatory loop in macrophage activation syndrome, and it has been shown to be associated with remarkable effectiveness and an excellent safety record.

## Clinical Syndrome

After an incubation period of 2–21 days, the symptoms of EVD present that resemble flu, including chills, fever, myalgia, and general malaise. As the disease progresses, abdominal complaints such as anorexia, nausea, vomiting, abdominal pain, diarrhea, as well lethargy, headache, and hypotension develop. Also bleeding may develop in 30–50% of patients, however, the severity varies. Bleeding is not *per se* associated with fatal outcome. In severe and fatal forms, death occurs between 6 and 16 days after symptoms appeared because of shock, seizures, severe metabolic complications, and bleeding. In lethal cases, a high blood viral load is seen, accompanied by a significant increment in blood neutrophils, drop in numbers of lymphocytes and platelets, and coagulation abnormalities (1).

Laboratory findings include initially leukopenia (which may drop to 1000 cells/μL) with lymphopenia, while neutrophilia develops thereafter with a left shift with atypical lymphocytes, thrombocytopenia (50000–100000 cells/μL), elevated liver functions with higher aspartate aminotransferase levels compared to alanine aminotransferase, hyperproteinemia, and proteinuria. Diffuse intravascular coagulopathy may be present because of prolonged prothrombin and partial thromboplastin times and the presence of fibrin split products. When a secondary bacterial infection eventually may develop, elevated numbers of white blood cells are documented. In fatal cases, a high blood viral load is seen, accompanied by a significant rise in blood neutrophil count, a progressive drop in lymphocyte and platelet counts, and diffuse coagulation abnormalities (1).

## Pathogenesis of Ebola Virus Diseases

Little is known about the pathology and pathogenesis of EVD in humans. Ebola virus has a broad cell tropism infecting not only immune cells like monocytes, macrophages, and dendritic cells but also various epithelial cells as well as endothelial cells, fibroblasts, hepatocytes, and adrenal cortical cells. Data from experimentally infected non-human primates using Zaire Ebola virus suggest that monocytes, macrophages, and dendritic cells are probably preferably infected at first, whereby these cells subsequently play a significant role to disseminate the virus by spreading it from the side of infection to draining lymph nodes and then to liver and spleen. The tropism of Ebola virus for lymphoid tissue, the liver, and the adrenal gland is important for the pathogenesis as adreno-cortical dysfunction by Ebola virus infection could contribute to the development of shock that characterizes the late stage of disease.

Studies in non-human primates and lethal human EVD show significant lymphocyte reduction and necrosis in spleen, thymus, and lymph nodes, although a significant cellular inflammatory reaction is lacking in these or other infected tissues. The apoptosis that is generally noticed in lymphocytes in infected humans and in non-human primates may clarify the increasing peripheral lymphopenia and tissue lymphoid depletion at post-mortem studies. T-lymphocyte and natural-killer cell populations are mostly reduced in macaques infected with Zaire Ebola virus (2) while human studies indicate that the number of NK cells generally decrease while fatal cases are characterized by especially reduced T-cells counts (3). In addition, blood d-dimer levels can be found in EVD, but are fourfold higher in non-survivors, indicating disseminated intravascular coagulation (DIC) as an important component of severe EVD (4).

## Host Immune Response during EVD

A substantial release of pro-inflammatory mediators and vasoactive elements has been shown in patients with severe EVD, contributing to inflammation and coagulation (5). In addition, an ineffective host response causes a high viral load that contributes to a pro-inflammatory state. The release of pro-inflammatory cytokines by monocytes and macrophages is deleterious by causing endothelial leakage, hypotension, DIC, and multiorgan failure, hence a syndrome that closely resembles septic shock. A recent study using 86 samples from the 2000–2001 Uganda outbreak correlated inflammatory biomarkers with clinical outcome, and found that death was associated with elevated concentrations of cytokines (IL-1α, IL-1RA, IL-6, MCP-1, MCSF, MIP-1α) and elevated d-dimer levels, as was found by others. Interestingly, this study also found elevated thrombomodulin and ferritin concentrations, being associated with hemorrhage and death (6).

Host immune responses have also been studied in non-human primates, the current gold standard experimental model of EVD. Strongly up-regulated cytokines/chemokines, including IL-1α, IL-1Ra, IL-6, MCP-1, MIP-1α, IL-15, and IL-18 were reported in experimentally infected macaques (7). Another striking finding was that, although initially fibrinogen levels increase, in the terminal stages of the infection plasma fibrinogen levels were found to decrease rapidly. In humans, plasma fibrinogen levels are generally low, as diffuse intravascular coagulation is very common (1).

## Hemophagocytic Lymphohistiocytosis

Hemophagocytic lymphohistiocytosis (also called macrophage activation syndrome) is an often fatal syndrome of exaggerated but ineffective inflammatory response, characterized by excessive macrophage and T-cell activation and an impaired capacity of NK cells and cytotoxic T cells to kill target cells (8). There are eight diagnostic criteria including fever, splenomegaly, cytopenias affecting at least two of the three lineages in the peripheral blood, hyperferritinemia >5000 μg/L, hypertriglyceridemia or hypofibrinogenemia, hemophagocytosis in the bone marrow, spleen, or lymph nodes, low or absent NK cell activity determined by 51-Cr release assay, and high levels of sCD25 (soluble interleukin-2 receptor). Five of these eight diagnostic criteria are required for diagnosis. Both familial (genetic due to mutations in the perforin pathway) (9) and acquired (reactive) forms of the syndrome are recognized, the latter being triggered by infectious, autoimmune, or neoplastic conditions. Most cases of virus-induced HLH are due to viruses of the herpes family, although a number of other viruses, including parvovirus B19, measles, mumps, influenza, enterovirus, and dengue have also been reported in association with HLH. The key event in HLH is a “cytokine storm” resulting in pancytopenia and hypertriglyceridemia. In addition, the macrophage activation leads to sustained and uncontrolled macrophage and histiocyte infiltration, resulting in hepatosplenomegaly, release of plasminogen activator promoting hypofibrinogenemia, up-regulation of hemoxygenase, and increase in ferritin levels.

## Hypothesis: Severe EVD is Characterized by Overwhelming Macrophage Activation Syndrome

Based on the clinical and pathophysiological characteristics of EVD, we hypothesize that infection with Ebola virus is associated with overwhelming macrophage activation in the setting of a secondary HLH. Strong arguments for this hypothesis are the presence of fever, cytopenia, hypofibrinogenemia, low NK cells, and strikingly elevated ferritin levels, which are also commonly found in EVD, especially in the patients with a fatal outcome. In addition, less perforin mRNA expression was found in peripheral blood mononuclear cells of patients with fatal Ebola infection compared to those that survived (10), while animal studies indicated that CD8-mediated protection against Ebola is perforin-dependent (11).

Cytopenias are key laboratory markers of HLH and are attributed to severe cytokine-mediated inflammation (12). Patients with Ebola infection are often leucopenic at presentation, with abnormal low numbers of lymphocytes and an increased percentage of granulocytes (13). Interestingly, total leukocyte numbers abnormally increase with a rise in immature granulocytes and atypical lymphocytes while the disease develops: this may mirror hyperactivation of bone marrow by cytokines of the IL-1 family that have been proposed to mediate the systemic hyperinflammatory reaction in HLH (14). Thrombocytopenia is a constant feature of EVD, with bone-marrow studies suggesting that thrombocytopenia is not due to reduced platelet production but rather to diffuse intravascular coagulation, as has also been reported in 40–90% of HLH cases (12).

A striking recent finding is the hyperferritinemia in Ebola patients, with mean values around 10,000 μg/L in non-survivors, and 2,500 μg/L in non-fatal cases at day 6–10 after symptom onset. Although hyperferritinemia can be encountered in infections, autoimmune diseases, and several rare syndromes, such very high ferritin concentrations of more than 10,000 μg/L indicate HLH with a sensitivity of 90% and specificity of 96% (ref).

There are also characteristics of EVD that might argue against a classical HLH syndrome. Histiocytic infiltrates of various organs, including liver and spleen, are to be expected in HLH; however, a recent review of human pathology studies indicates that only minimal inflammation is observed in infected tissue and organs in fatal cases, despite high viral loads and necrotic lesions (5). However, Kupffer cell hyperplasia is seen in Ebola infections, similar to HLH (15). Furthermore, no hemophagocytosis has been reported to date in EVD, although very few (if any) studies have been done in this respect. Moreover, the hemophagocytic activity might not occur at all stages of the disease (12) and therefore does not have to be present to diagnose HLH (8).

## Conclusion and Future Perspectives

In conclusion, the clinical manifestations and pathophysiology of severe EVD lead us to propose that HLH characterized by overwhelming macrophage activation is a key event in the pathogenesis of the Ebola infection.

It has been proposed that sepsis, systemic inflammatory response syndrome, multiple organ dysfunction syndrome, and HLH form a disease continuum that share a common mechanism: systemic immune dysregulation triggered by a specific external agent (12). We think that these conditions occur in Ebola virus infection. The metabolic and immune imbalance during EVD requires both optimal support measures and the removal of the trigger (infection) that stimulate inflammation. Unfortunately, the latter is not feasible, because no antiviral medication has been of proven efficacy in EVD.

The identification of HLH as part of severe EVD, however, brings the possibility for a pathophysiologically targeted approach of therapy, using the arsenal of immunomodulatory medication known to be successful in HLH. While plasma exchange, corticosteroids, and even immunosuppressive therapy have been proposed for treatment of HLH, special promise is represented by cytokine-directed therapy in the form of recombinant interleukin-1 receptor antagonist (anakinra). Treatment with anakinra has the advantage to interrupt the deleterious IL-1-mediated hyperinflammatory loop in macrophage activation syndrome, and it has been shown to be associated with remarkable effectiveness (16–20). Moreover, the excellent safety record of anakinra argues for its compassionate use in patients with severe EVD in humans.

## Author Contributions

All authors contributed substantially to the present work, revised the work critically, approved the final paper, and agreed to be accountable for all aspects of the work.

## Conflict of Interest Statement

The authors declare that the research was conducted in the absence of any commercial or financial relationships that could be construed as a potential conflict of interest.

## References

[1] Feldmann H Geisbert TW. Ebola haemorrhagic fever. Lancet (2011) 377(9768):849–62. 10.1016/S0140-6736(10)60667-8 21084112 PMC3406178

[2] Geisbert TW Young HA Jahrling PB Davis KJ Larsen T Kagan E Pathogenesis of Ebola hemorrhagic fever in primate models: evidence that hemorrhage is not a direct effect of virus-induced cytolysis of endothelial cells. Am J Pathol (2003) 163(6):2371–82. 10.1016/S0002-9440(10)63592-4 14633609 PMC1892396

[3] Sanchez A Lukwiya M Bausch D Mahanty S Sanchez AJ Wagoner KD Analysis of human peripheral blood samples from fatal and nonfatal cases of Ebola (Sudan) hemorrhagic fever: cellular responses, virus load, and nitric oxide levels. J Virol (2004) 78(19):10370–7. 10.1128/JVI.78.19.10370-10377.2004 15367603 PMC516433

[4] Rollin PE Bausch DG Sanchez A. Blood chemistry measurements and d-Dimer levels associated with fatal and nonfatal outcomes in humans infected with Sudan Ebola virus. J Infect Dis (2007) 196(Suppl 2):S364–71. 10.1086/520613 17940972

[5] Martines RB Ng DL Greer PW Rollin PE Zaki SR. Tissue and cellular tropism, pathology and pathogenesis of Ebola and Marburg viruses. J Pathol (2014) 235(2):153–74. 10.1002/path.4456 25297522

[6] McElroy AK Erickson BR Flietstra TD Rollin PE Nichol ST Towner JS Ebola hemorrhagic fever: novel biomarker correlates of clinical outcome. J Infect Dis (2014) 210(4):558–66. 10.1093/infdis/jiu088 24526742 PMC4172044

[7] Ebihara H Rockx B Marzi A Feldmann F Haddock E Brining D Host response dynamics following lethal infection of rhesus macaques with Zaire Ebola virus. J Infect Dis (2011) 204(Suppl 3):S991–9. 10.1093/infdis/jir336 21987781 PMC3189992

[8] Rosado FG Kim AS. Hemophagocytic lymphohistiocytosis: an update on diagnosis and pathogenesis. Am J Clin Pathol (2013) 139(6):713–27. 10.1309/AJCP4ZDKJ4ICOUAT 23690113

[9] Terrell CE Jordan MB. Perforin deficiency impairs a critical immunoregulatory loop involving murine CD8(+) T cells and dendritic cells. Blood (2013) 121(26):5184–91. 10.1182/blood-2013-04-495309 23660960 PMC3695362

[10] Baize S Leroy EM Georges-Courbot MC Capron M Lansoud-Soukate J Debre P Defective humoral responses and extensive intravascular apoptosis are associated with fatal outcome in Ebola virus-infected patients. Nat Med (1999) 5(4):423–6. 10.1038/7422 10202932

[11] Gupta M Greer P Mahanty S Shieh WJ Zaki SR Ahmed R CD8-mediated protection against Ebola virus infection is perforin dependent. J Immunol (2005) 174(7):4198–202. 10.4049/jimmunol.174.7.4198 15778381

[12] Ramos-Casals M Brito-Zeron P Lopez-Guillermo A Khamashta MA Bosch X Adult haemophagocytic syndrome. Lancet (2014) 383(9927):1503–16 10.1016/S0140-6736(13)61048-X 24290661

[13] Kortepeter MG Bausch DG Bray M. Basic clinical and laboratory features of filoviral hemorrhagic fever. J Infect Dis (2011) 204(Suppl 3):S810–6. 10.1093/infdis/jir299 21987756

[14] Ravelli A Grom AA Behrens EM Cron RQ. Macrophage activation syndrome as part of systemic juvenile idiopathic arthritis: diagnosis, genetics, pathophysiology and treatment. Genes Immun (2012) 13(4):289–98. 10.1038/gene.2012.3 22418018

[15] Tsui WM Wong KF Tse CC Liver changes in reactive haemophagocytic syndrome. Liver (1992) 12(6):363–7 10.1111/j.1600-0676.1992.tb00590.x 1470007

[16] Bruck N Suttorp M Kabus M Heubner G Gahr M Pessler F. Rapid and sustained remission of systemic juvenile idiopathic arthritis-associated macrophage activation syndrome through treatment with anakinra and corticosteroids. J Clin Rheumatol (2011) 17(1):23–7. 10.1097/RHU.0b013e318205092d 21169853

[17] Butin M Mekki Y Phan A Billaud G Di Filippo S Javouhey E Successful immunotherapy in life-threatening parvovirus B19 infection in a child. Pediatr Infect Dis J (2013) 32(7):789–92. 10.1097/INF.0b013e31828df4d1 23429554

[18] Kahn PJ Cron RQ Higher-dose Anakinra is effective in a case of medically refractory macrophage activation syndrome. J Rheumatol (2013) 40(5):743–4 10.3899/jrheum.121098 23637382

[19] Loh NK Lucas M Fernandez S Prentice D. Successful treatment of macrophage activation syndrome complicating adult Still disease with anakinra. Intern Med J (2012) 42(12):1358–62. 10.1111/imj.12002 23253002

[20] Rajasekaran S Kruse K Kovey K Davis AT Hassan NE Ndika AN Therapeutic role of anakinra, an interleukin-1 receptor antagonist, in the management of secondary hemophagocytic lymphohistiocytosis/sepsis/multiple organ dysfunction/macrophage activating syndrome in critically ill children*. Pediatr Crit Care Med (2014) 15(5):401–8. 10.1097/PCC.0000000000000078 24583503 PMC7122718

